# Is oral lichen planus a risk factor for peri-implant diseases? A systematic review and meta-analysis

**DOI:** 10.1186/s12903-020-01134-2

**Published:** 2020-05-20

**Authors:** Xiaoqin Xiong, Tiantian Xu, Xinhong Wang, Wenguang Qin, Ting Yu, Gang Luo

**Affiliations:** Department of Periodontology and Oral Medicine, Key Laboratory of Oral Medicine, Guangzhou Institute of Oral Disease, Stomatology Hospital of Guangzhou Medical University, NO.195 Dongfeng West Road, Guangzhou, 510140 China

**Keywords:** Dental implants, Peri-implant diseases, Oral lichen planus, Systematic review, Meta-analysis

## Abstract

**Background:**

To evaluate whether oral lichen planus (OLP) is a risk factor for peri-implant diseases (PIDs) with a systematic review and meta-analysis.

**Methods:**

Five electronic databases including Medline, Embase, Web of Science, the Cochrane Library and Scopus were searched. The included studies are observational human studies written in English. The population of interest included those with/without OLP who received dental implant treatment. The follow-up time after implantation was from 1 month to 20 years. The quality of the included articles regarding risk of bias and methodology were assessed with the Newcastle-Ottawa Scale or the Agency for Healthcare Research and Quality. The data involving exposure (OLP), primary outcomes (implants having PIDs) and secondary outcomes (probing depth/PD, bleeding on probing/BOP and bone loss/BL) and potential confounders were extracted. Heterogeneity was assessed by I^2^ test. Dichotomous data are expressed as the risk ratio (RR) and 95% confidence interval (CI) which were calculated with a fixed effect model.

**Results:**

Of the 66 articles, two studies were enrolled and evaluated as high quality, which totally contained 68 participants receiving 222 (OLP vs. non-OLP, 112 vs. 110) implants with 12 to 120-month follow-up time. Proportions of implants with PIDs between OLP and non-OLP groups were as follows: 19.6% (22/112) vs. 22.7% (25/110) for PIM and 17.0% (19/112) vs. 10.9% (12/110) for PI. The meta-analysis revealed no recognizable difference in number of implants with PIDs (PI: RR = 1.49, 95% CI 0.77–2.90, *P* = 0.24; PIM:RR = 0.88, 95% CI 0.53–1.46, *P* = 0.61; PIDs: RR = 1.08, 95% CI 0.75–1.55, *P* = 0.68) or BOP (RR = 0.90, 95% CI: 0.70–1.15, *P* = 0.40) between OLP and non-OLP groups.

**Conclusions:**

Available articles regarding the effects of OLP on PIDs remains very limited. Existing evidence does not support OLP as a suspected risk factor for PIDs. Large-scale prospective trials are required to validate the findings.

## Background

Dental implant treatment is a well-established method for restoring lost teeth. Despite a high rate of survival (> 94%) for more than 5 years [1, 2], a very high proportion (8–44%) of dental implants develop peri-implant diseases (PIDs), including peri-implant mucositis (PIM) and peri-implantitis (PI) [3], which can lead to implant failure or loss. PIDs are pathological inflammatory conditions, of which PIM involves only peri-implant mucosal inflammation, while PI causes both peri-implant mucosa and alveolar bone damage [4], accompanied by bleeding on probing, suppuration, increased probing depth, and progressive marginal bone loss [5]. PID-associated risk factors include poor oral hygiene [6], smoking [7], a history of periodontitis [4, 8] and systemic diseases, such as diabetes [9].

Oral lichen planus (OLP), a chronic systemic autoimmune disease, affects one to 2 % of the general population, especially middle-aged and elderly females [10]. Typically, OLP manifests as plaque-like or white reticular lesions, an erythematous area or ulceration [11, 12], and it can affect the oral mucosa, tongue or gingiva [13]. Recently, OLP has been questioned to be a potential risk indicator for PIDs. OLP patients have been found to have poor oral hygiene [14] and quality of life [15]. Furthermore, some studies have shown a very high implant failure rate (42/55) for OLP patients receiving implant placement during the acute stages [16], some researchers have also demonstrated that OLP patients feel more stress and have a weaker psychological profile [17]. These concerns have previously made some dentists consider OLP a contraindication to implant treatment. However, some studies, in which 66 short implants were implanted in 23 OLP patients with a survival rate of 98.5%, did not observe any difference in the success rate between OLP patients and normal controls [18, 19]. Notably, most of the studies regarding implantation in OLP populations were observational studies (including case reports, retrospective studies, etc.) with small sample sizes. Hence, whether dental implantation in OLP patients is safe remains inconclusive. The present systematic review aimed to analyze the existing articles to determine whether OLP is a risk factor for PIDs.

## Methods

### Focused question

Compared with non-OLP populations, are there changes in the rates of probing depth, bone loss or bleeding on probing around implants in OLP patients? Are there different rates of PIM and PI prevalence in OLP patients compared with non-OLP populations?

This manuscript has been prepared based on the Preferred Reporting Items for Systematic Review and Meta-analyses (PRISMA) guidelines for reporting systematic reviews.

### Population and exposure

The population of interest was patients with and without OLP who received dental implant treatment. The studies included participants aged ≥18 years with no periodontitis or stable periodontitis after treatment. Their OLP status was diagnosed before implant treatment. It was preferred that OLP patients had a diagnosis of the OLP subtype or concomitant symptoms, such as desquamative gingivitis which could be considered separate exposure factors. Detailed records of treatments, including medications, were included for all OLP patients. The primary outcomes were PIDs, and the secondary outcomes were probing depth, bleeding on probing, and bone loss. Implant failure was also considered if its infective origin was clearly stated.

### Disease determinants, risk factors, and etiologic agents

The potential association of OLP with PID determinants, including sex, age, educational background, socioeconomic position, genetics, lifestyle, nutrition, health behaviors, and microbiological factors, was recorded when available. Whenever possible, the quality of the determinant/exposure measurements was assessed.

There are some general risk indicators/factors for PI and PIM, including a history of periodontitis [20], systemic diseases, genetic traits, and smoking. Local risk indicators/factors for PI and PIM include oral hygiene/poor plaque control, poor compliance with supportive implant therapy, presence of excess cement, implant materials and surface characteristics [21], design of implant-supported prostheses and dimensions of keratinized mucosa [4, 8].

### Study follow-up duration

All studies with a mean duration or follow-up interval of at least 1 month were included because the earliest time for PID diagnosis was approximately 1 month after loading restorations [4, 22]. If the follow-up time was reported as a range without means or medians, the minimum should be no less than 1 month.

### Types of studies

With the goal of identifying studies for inclusion, certain methods were designed based on acceptability criteria in this study. Eligible studies, including observational or longitudinal studies or prospective research, were those with a follow-up time ≥ 1 month, with primary outcomes of PIDs and implants loss or failure. The secondary outcomes were probing depth, bleeding on probing and bone loss.

### Inclusion criteria

1. Observational human studies, such as case-control, cross-sectional, retrospective or prospective cohort studies;
2. Studies in which OLP and PIDs were the main exposure factor and outcome, respectively;
3. Both OLP and PIDs should be definitely diagnosed in studies, the diagnosis of OLP was made according to clinical and pathological evidence, the diagnosis of PIDs based on clinical settings, soft tissue inflammation was detected by detecting BOP, BL should be confirmed by PD and radiographic examinations.
4. Studies with a follow-up time of ≥1 month after implant placement; and
5. Studies published in the English language.

### Exclusion criteria

1. Studies lacking control groups (i.e., a non-OLP group);
2. Narrative reviews, comments, letters, case reports or series, and conference abstracts; and
3. Duplicates were removed, and the latest or most complete literature was retained.

### Search strategy

The search was performed in June 2019. All available articles published online before this time in five electronic databases, including Medline, Embase, Web of Science, the Cochrane Library and Scopus, were retrieved. Manual searching in dental journals, especially those focusing on implantology, was also performed. The search strategy was established with adequate discussion among all the coauthors (Table 1).

**Table 1:** Electronic databases used and search strategies

	Database/journal	Search strategy	Items found
Electronic searching	Medline	#1 [All Fields] AND #2 [All Fields] AND #3[All Fields]	24
	Embase	#1:ti, ab, kw AND #2:ti, ab, kw AND #**3**:ti, ab, kw	10
	Web of Science	TS = *#1* AND TS = *#2* AND TS = *#3*	11
	Cochrane Library	#1:ti, ab, kw AND #2:ti, ab, kw AND #3:ti, ab, kw	1
	Scopus	TITLE-ABS-KEY (#1) AND TITLE-ABS-KEY (#2) AND TITLE-ABS-KEY (#3)	11
Manual searching	Journal of Periodontology	#1 AND #2 AND #3	1
	Clinical Implant Dentistry and Related Research	#1 AND #2 AND #3	3
	Clinical Oral Implants Research	#1 AND #2 AND #3	1
	Journal of Clinical Periodontology	#1 AND #2 AND #3	1
	British Journal of Oral & Maxillofacial Surgery	#1 AND #2 AND #3	1
	Clinical Oral Investigations	#1 AND #2 AND #3	1
	Journal of Dentistry	#1 AND #2 AND #3	1

#1: “dental implant” OR “implant”; #2 “peri-implantitis” OR “peri-implant disease” OR “peri-implant mucositis” OR “loss” OR “failure” OR “success rate” OR “survival rate”; #3 OLP OR “oral planus lichen”

### Study selection

Two researchers (XX and XT) screened the articles via abstract and full-text review independently and in duplicate. Afterward, articles that met the research topic were enrolled for full-text screening. If an article could not be excluded/included based on its title and abstract, it will be also subject to full-text review. Subsequently, the articles were independently assessed in duplicate by the reviewers. Any disparity about research eligibility was settled by discussion among the two reviewers, and if no consensus could be reached, the decision was resolved through arbitration by a third reviewer (YT). All articles that did not fulfill the eligibility criteria were excluded.

For studies with missing data or those for which a clear decision on study eligibility could not be made after assessment of the full text, the corresponding authors were e-mailed to ask for the information needed for a final decision. Messages via social media or academic websites were also sent to the corresponding authors in the absence of response by e-mail.

### Data extraction and management

A detailed form designed specifically for data extraction was used, and all the relevant data from the collected articles were independently extracted by two independently. Any disagreements were settled through evaluation by a third examiner (YT) or group consensus.

The following research information was extracted (Table 2):

1. Type of research;
2. Sample framework (e.g., community, university);
3. Sample size, sex and age of participants;
4. Diagnostic criteria for PI, PIM and OLP;
5. Follow-up time; and
6. Confounding factors relative to exposure factors.

**Table 2:** General description of the included studies

Study	Gonzalo Hernandez (2012)	Pia López-Jornet (2014)
Age (year)	Median (range) 53.7 (38–73)/52.2 (35–70)	Median (range) 64.5 (44–76)/42 (29–79)
OLPG/ CG	18/18	16/16
Male/Female	10/26	14/18
No. of implants	56/60	56/50
Definition of OLP	Clinical and histopathological criteria of OLP according to the modified WHO diagnostic criteria of OLP	OLP was diagnosed based on a thorough clinical examination and histopathology of the lesions
Definition of PIM and PI	The presence of PIM (BOP, PD ≥ 4 mm and no BL) and PI (BOP or pus, BL ≥ 3 threads at the final examination)	Diagnosis of PIDs based on clinical indicators (e.g. CAL, PD, BL)
Follow-up time (months)	Median (IR): 56.5 (22)/52.5 (22.7)	Median (range): 42 (12–120)/48 (24–48)
PD (mm):	PD (mm): n:< 4 mm 23/18; ≥4 mm 33/42	PD (mm): median (range): 3.00 (1.12–4.90)/3.00 (2–5)
BOP	(sites): 105/114; (implants): 36/44; patients: 13/16	(No. implants): 12/11
BL	BL (mm): ≤1.7 18/22; 1.8–2.4 24/24; 2.5–3 8/9; 3.1–3.6 4/3; ≥3.7:2/2	(No. of implants): 10/8;
PIM (No. of implants)	12/16	10/9
PI (No. of implants)	5/4	14/8
Confounders controlled for	Smoking	Age, sex, smoking, alcohol consumption, frequency of tooth brushing
Key findings	Lichen planus was not a prominent local factor in the genesis of implant failure.	Implants did not influence manifestations of OLP. OLP was not a risk factor for peri-implantitis.
Odds Ratio	_	1.32 (PI)
95% Confidence Interval	_	0.81–2.14 (PI)
*p* Value	.254 (PI)/.985 (PIM)	.257 (PI)

*M/F* male/female, *OLPG* oral lichen planus group, *CG* control group, *BL* bone loss, *BOP* bleeding on probing, *CAL* clinical attachment level, *PD* probing depth, *PI* peri-implantitis, *PIM* peri-implant mucositis, *S* systemic diseases

### Outcomes

1. Probing depth change;
2. Bone loss level change;
3. Bleeding on probing level change;
4. Number of implants with/without PI and PIM; and
5. Number of implants loss or failure.

### Quality assessment

The Newcastle Ottawa Scale (NOS) [23] and the Agency for Healthcare Research and Quality (AHRQ) [24] standards were used to assess the quality of cohort and cross-sectional studies respectively. The cohort study degree of bias was categorized with a star-based quality assessment system that considers three categories as follows: the selection of the study groups (four stars), the comparability of the groups (two stars), and the ascertainment of either the exposure or outcome of interest (three stars). An 11-item checklist recommended by the AHRQ was used to assess the methodological quality of the cross-sectional studies. Article quality was assessed as follows: low quality (0 to 3); moderate quality (4 to 7); and high quality (8 to 11).

### Data synthesis

Statistical heterogeneity in risk in patients suffering from PIDs in the collected studies was evaluated using both risk ratios (RRs) and I^2^ measures. The use of I^2^ values was based on the Cochrane Handbook: 0 < I^2^ < 40% represents a lack of heterogeneity; 30% < I^2^ < 60% represents moderate heterogeneity; 50% < I^2^ < 90% signals substantial heterogeneity; and 75% < I^2^ < 100% shows considerable heterogeneity.

When suitable, meta-analysis was used to assess risk in patients suffering from PIDs based on implant and patient characteristics. A random effects approach was used if there was mild to moderate statistical heterogeneity, otherwise, a fixed-effect approach was chosen. Considering that only observational studies were included in the present systematic review, the primary outcome variable was the RRs of PI and PIM. The RRs were calculated with 95% confidence intervals (CIs) (Review Manager, v5.2, The Nordic Cochrane Center, The Cochrane Collaboration, Copenhagen, Denmark) to quantify the association between OLP and the risk of PI and PIM. Forest plots were generated showing the RRs and 95% CIs of the involved studies. If there were more than 10 studies included in the meta-analysis, publication bias was evaluated qualitatively by a funnel plot [25].

## Results

### Search

**Fig. 1:**
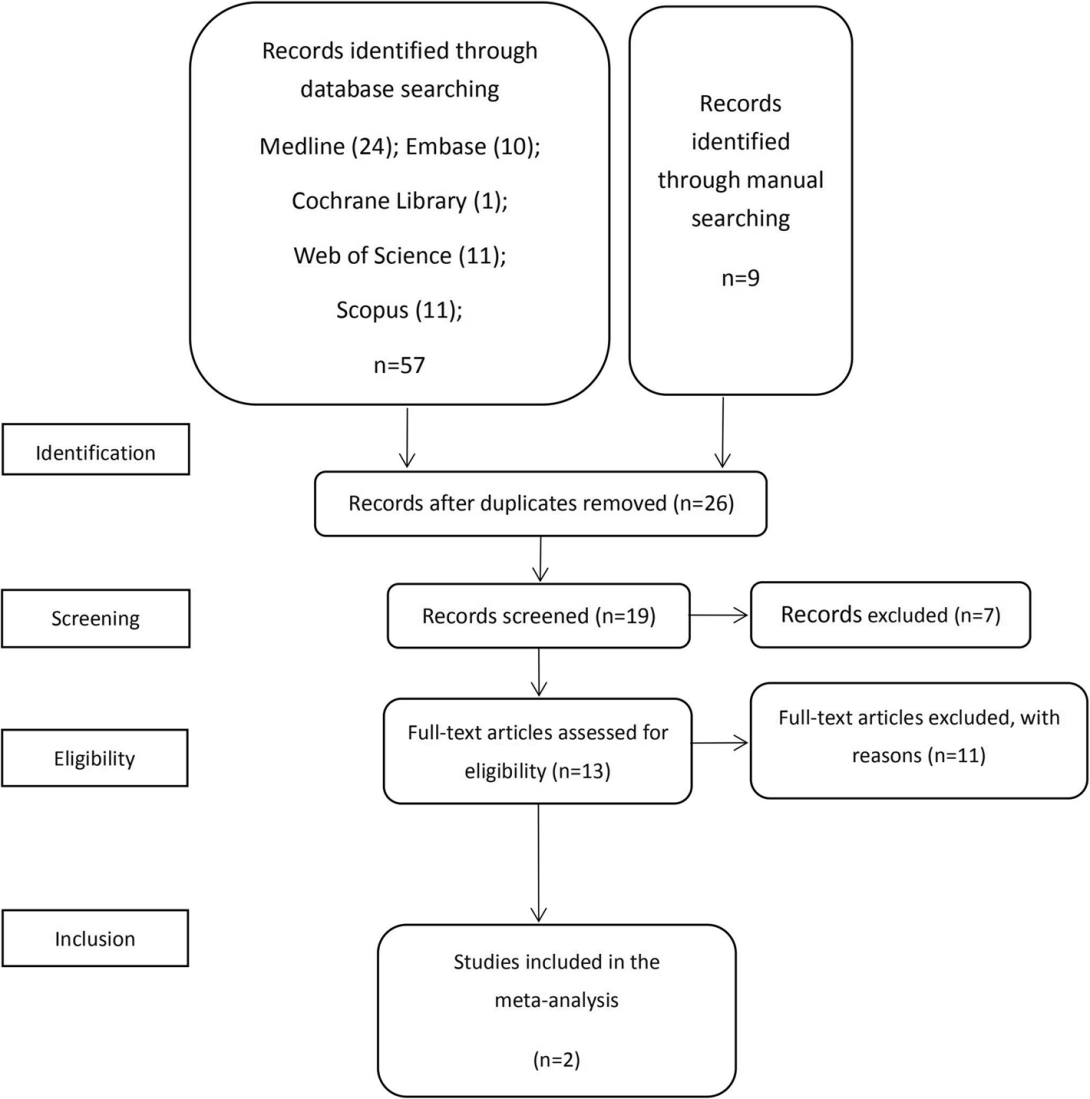
Study screening process

A total of 66 studies were identified after electronic and manual searches (Fig. 1)**.** After removing duplicates, 26 articles were retained. Then, 7 articles were excluded after evaluating the titles and abstracts, and the remaining 19 articles were selected for full-text evaluation. Of the 19 articles, 13 articles involved both OLP and implant treatment. Then, 11 articles were excluded for various reasons, such as being case reports, reviews, etc., and the remaining 2 articles were retained for final review. According to the Inclusion and exclusion criteria, there was no studies available on implant loss or failure as the primary outcome. The screening outcomes were similar regardless of whether the article was published in English [26].

### Study characteristics

#### Location and sample characteristics

The two included studies included one prospective study and one cross-sectional study [15] (Table 2). Both studies were conducted in the same country (Spain) but by different institutions. The two studies contained 34 OLP patients and 34 non-OLP controls aged between 29 and 79 years. 68 participants received 224 implants (OLP vs. non-OLP, 112 vs. 110) with a follow-up time of 12 to 120 months.

At the implant level, 19.6% (22/112) and 22.7% (25/110) developed PIM in the OLP and non-OLP groups, while 17.0% (19/112) and 10.9% (12/110) developed PI in the two groups, respectively. Additionally, bleeding on probing was present at the sites of 42.6% (48/112) and 50.0% (55/110) of implants in the two groups, respectively.

The ratios of PIDs in two groups could not be calculated at the individual level due to the missing data. The outcomes regarding probing depth and bone loss could not be combined because they were reported in different forms, and only one study’s authors provided the original data for these parameters by online contact.

#### Risk of bias and methodological quality

Quality evaluation according to the NOS showed that the prospective study was rated as having 7 stars and was classified as high quality (Table 3). Based on the AHRQ criterion, the final score of the cross-sectional study was 8, and it was classified as having high quality (Table 4).

**Table 3:** Quality assessment of the prospective study

Study	Selection (max 4 asterisks)	Comparability (max 2 asterisks)	Exposure (max 3 asterisks)	Score	Quality
Gonzalo Hernandez (2012)	***	**	**	7	Low risk

**Table 4:** Quality assessment of the cross-sectional study

Items	Yes	No	Unclear
1) Define the information (survey, record review)	1		
2) List inclusion and exclusion criteria for exposed and unexposed subjects (cases and controls) or refer to previous publications	1		
3) Indicate time period used for identifying patients	1		
4) Indicate whether or not subjects were consecutive if population-based	1		
5) Indicate if evaluators of study subjects were blinded to other aspects of the status of the participants	1		
6) Describe any assessments undertaken for quality assurance purposes (e.g., test/retest of primary outcome measurements)	1		
7) Explain any patient exclusions from analysis			0
8) Describe how confounding was assessed and/or controlled	1		
9) If applicable, explain how missing data were handled in the analysis			0
10) Summarize patient response rates and completeness of data collection			0
11) Clarity what follow-up, if any, was expected and the percentage of patients for which incomplete data or follow-up was obtained	1		

#### Risk of PIDs in the OLP versus non-OLP groups

**Fig. 2:**
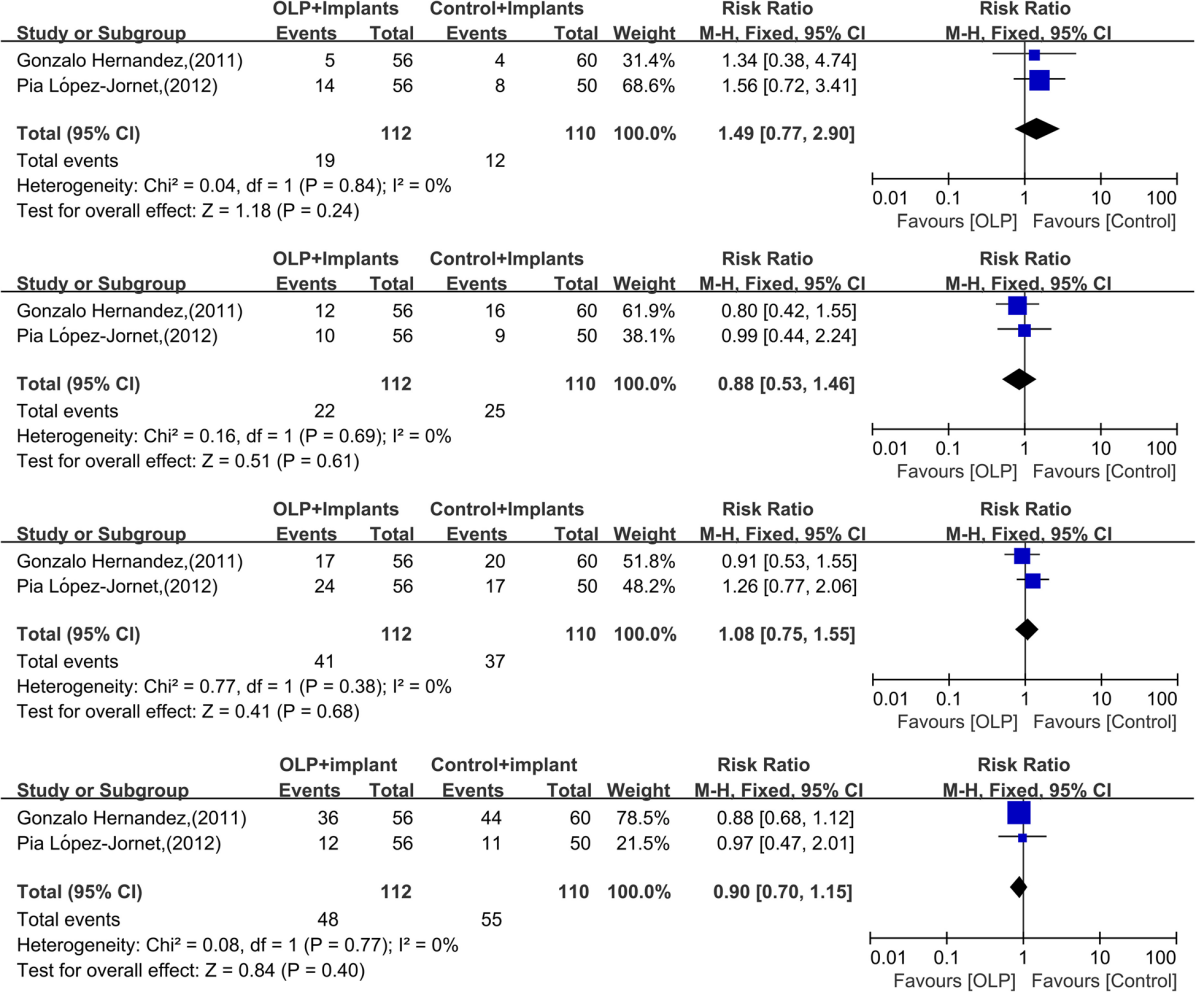
Forest plot of the meta-analysis of PI **a**, PIM **b**, PIDs **c** and BOP **d**. #CI, confidence interval

Due to the small sample size and good homogeneity (χ^2^ = 0.04, *P* = 0.84; *I*^2^ = 0) of the included articles, the fixed-effect model was employed to calculate the pooled RR in this meta-analysis. The model showed no significant difference in the number of implants suffering from PI (*RR* = 1.49, 95% *CI*: 0.77–2.90, *P* = 0.24, Fig. 2a) or PIM (*RR* = 0.88, 95% *CI*: 0.53–1.46, *P* = 0.61, Fig. 2b) between the OLP and non-OLP groups at the implant level. There was a similar result when combining PI and PIM into PID (*RR* = 1.08, 95% *CI*: 0.75–1.55, *P* = 0.68, Fig. 2c). This was also the case for the presence of bleeding on probing between the two groups (*RR* = 0.90, 95% *CI*: 0.70–1.15, *P* = 0.40, Fig. 2d).

## Discussion

### Key findings

The present systematic review assessed the current existing evidence on the relationship between OLP and PIDs for the first time and attempted to determine whether OLP is a potential risk factor for PIDs. Of 139 studies, two, which contained 68 participants receiving 222 (OLP vs. non-OLP, 112 vs. 110) implants, with a 12- to 120-month follow-up time, were included and evaluated as having high quality. The proportions of implants with PIDs between the OLP and non-OLP groups were as follows: 19.6% (22/112) vs. 22.7% (25/110) for PIM and 17.0% (19/112) vs. 10.9% (12/110) for PI. Existing evidence seems to suggest that OLP is not a suspected risk factor for PIDs during the 10-year follow-up period.

### Overall completeness and applicability of the evidence

The diagnostic criteria for OLP were clearly stated in the two enrolled studies, while those for PIDs were absent in the cross-sectional study. Moreover, in the two enrolled studies, PIDs were evaluated at only the implant level and not at the individual level, while OLP was diagnosed at the individual level. Hence, the evidence was insufficient to determine whether OLP is a risk factor for PIDs at the individual level.

Concerning secondary outcomes of PIDs, both studies had complete bleeding on probing data. After data combination, OLP was found to not be a risk factor for bleeding on probing at the implant level. For the secondary outcomes for PIDs, i.e., probing depth and bone loss, the data were complete but reported in different forms in the two studies. The prospective study set cutoff values for probing depth and bone loss to determine the percentages of involved implants, while the cross-sectional study directly presented the medians and ranges of the two parameters. Despite repeated contact with the authors, we obtained only the raw data of the prospective study. Hence, an attempt to combine the data of the two studies in terms of probing depth or bone loss could not be achieved. Ultimately, both studies found no significant difference in probing depth or bone loss between the OLP and non-OLP groups.

When the participant was used as the unit of analysis, the intragroup analysis of the OLP group failed to demonstrate a statistically significant relationship between the existence of desquamative gingivitis and PIM or desquamative gingivitis and PI, but 20 out of 25 implant patients with PIM were among those with desquamative gingivitis in the OLP group; hence, PIM with DG in the OLP group occurred more frequently than non-desquamative gingivitis in the OLP group in this subgroup of implant patients in the prospective study [27]. Since the cross-sectional study presented insufficient information, although the corresponding authors were contacted in various ways and only the author of the prospective study provided the original data of the parameters of interest, no subgroup analysis was performed. Therefore, subgroup combinations in the meta-analysis were not possible. We considered implant failure as an outcome but get no eligible article. Five articles were excluded in the screening step due to case reporting, no control group, etc. Despite no parallel studies comparing implant failure between OLP patients and non-OLP controls, OLP patients have shown a similar implant failure rate (2.7%, 14/523) to populations without OLP (~ 2%) [28].

### Overall quality, strength, and consistency of the evidence

The present systematic review included only two observational studies that met the inclusion criteria mainly because related research on implantation in OLP populations is scarce. However, the durations of the two enrolled studies were very similar (2003 to 2009 vs. 2005 to 2010), and the study populations were from the same country, which may be beneficial for data synthesis. Nevertheless, the results might be more applicable to specific ethnicities or countries and may not be easily generalized for people globally. The average age of the OLP patients in the two studies was quite different (64.5 vs. 53.7). This distinction may introduce uncertainty regarding the applicable age after pooling the data. Additionally, the mean follow-up time of the two studies was similar but did not exceed 5 years; therefore, it is unclear whether OLP is a risk factor for PIDs over an observation period longer than 5 years. The two studies contained detailed records of important confounders, such as smoking, but only the prospective study controlled for confounders. The response rate was not reported in either study; thus, the number of lost samples was unknown. In the prospective study, two implants in the non-OLP group were lost for unknown reasons, and the study did not state whether it was a PI-associated consequence. Even if that was the case, there was no difference in the results of the PI (*RR* = 1.31, 95% *CI*: 0.70–2.45, *P* = 0.40) analysis between the two cases.

Case definitions for PIDs vary considerably in previous studies [8], and PID data were especially challenging to interpret. Only the prospective study, which reported clear definitions of PIM and PI, was evaluated according to the defined criteria. The cross-sectional study did not specify the diagnostic criteria of PIDs in detail, while the clinical indicators (e.g. BOP, PD, BL) were described in detail with table. The two studies had different definitions of diseases, and there may have been some bias in the data. The diagnostic criteria for PI in the study were not uniform, and limited studies were included in the meta-analysis, so no meta-analysis could be conducted to prove the effect of the differences on the meta-analysis outcomes. Clinical indicators such as bleeding on probing and bone loss are relatively easy to standardize, although no effect of OLP on these indicators has been observed. Implant position has been considered as a prognostic factor for implant success [29]. However, we could not perform subgroup analysis with implant position as a grouping variable due to lack of raw data. Anyway, implants from the two studies showed equal distributions in jaws (maxilla/mandible: 52/60 vs. 63/49, χ^2^ = 2.162, *P* = 0.141) and tooth sites (20/92 vs. 23/89, χ^2^ = 0.259, *P* = 0.611) between OLP and non-OLP groups, suggesting implant position might be not a source of heterogeneity to affect the conclusions.

The results of the statistical analysis show that the homogeneity between the results was good. However, the limited number of enrolled studies made it unfeasible to draw a funnel plot to assess the variation in the studies. Although the sample size was small, the strength of the study evidence was high as analyzed by quality-control assessment, and the overall estimate of the meta-analysis represents the best available evidence.

### Implications for practice and policy

Although there was no clear association between OLP and the occurrence of PIDs, implant-related issues in OLP patients cannot be easily ignored, and several issues are worth noting. First, in a clinical study [14], the gingival index of OLP group was calculated, and the mean value of periodontal index and rate of bleeding on probing were higher than those in control group, revealing that the periodontal condition in the OLP group was poor compared with that in the control group. Poor oral hygiene is a well-known risk factor for PIDs, therefore, OLP may have a certain impact on PIDs. Second, some scholars believed that the capacity of epithelium to adhere to the titanium surface of the implants in OLP patients may be affected because the adhesion of epithelial cells decreases, affecting the epithelial barrier around the implant surface [30]. Third, desquamative gingivitis is a special type of OLP that causes gingival damage. An increased frequency of PIM was reported in cases of desquamative gingivitis compared with non-desquamative gingivitis OLP patients [27]. Another study demonstrated that the presence of desquamative gingivitis was associated with relatively deep periodontal pockets [31] . The study inferred that desquamative gingivitis lesions might circuitously enhance the long-term risk for periodontal diseases via plaque accumulation by impeding proper oral hygiene around natural teeth and implants. It is important to clarify the constructive associations between desquamative gingivitis and PIDs in the future. Moreover, OLP is a T cell-mediated chronic inflammatory autoimmune disease [32]. PIDs in humans cause leukocytic infiltration through barrier epithelial migration and an increase in the proportion of T and B cells [33]. In this context, these two conditions might aggravate each other via inflammatory pathways. Additionally, dysbiosis might be a potential link between OLP and PIDs, given that both conditions have been related to microbial alterations [34, 35] . Finally, systemic corticosteroid treatment in OLP patients was frequently associated with decreased bone mineral density, especially during the first 6 months of corticosteroid therapy [36]. The osteoporotic effects of corticosteroids might cause more alveolar bone loss around dental implants during infection [37].

In this systematic review, the available evidence did not support OLP as a risk factor for PIDs. However, this does not mean that implant restoration can be performed indiscriminately in OLP patients. For patients with acute/erosive forms of OLP or desquamative gingivitis, immune system disorder and poor oral hygiene may be potential risk factors for PIDs. In clinical settings, whether OLP is well-controlled or not would be a concern during implant treatment planning. The two studies included in the current review had declared that implantation therapy was conducted during the remission stages of OLP. Some study has shown a very high implant failure rate (42/55) for OLP patients receiving implant placement during the acute stages [16]. Reversely, controlled OLP patients and healthy condition have displayed an equal marginal bone loss around implant in four-year follow up [38]. Obviously, selecting the remission stages are clinically appropriate and normative. OLP should not be considered as a contraindication for implant treatment in patients who do not have evident symptoms or mucosal erosive congestion and have good oral hygiene. Furthermore, the mental state of OLP patients might be improved after implant treatment, which in turn is better for OLP control, considering that those without implant treatment have been reported to have a poor quality of life, a weaker psychological profile and greater stress [15, 17]. Additionally, for OLP patients receiving glucocorticoid treatment, assessment of alveolar bone mineral density in the area to be implanted should be considered. Finally, it is worth noting the effect of implants in mouth on the recovery of OLP patients, although there were no significant differences in OLP signs and symptoms between the implant group (14 patients) and nonimplant group (15 patients) during the 12–24 month follow-up period [39]. For OLP patients who have undergone implant restoration, OLP conditions may need to be monitored frequently, and implant maintenance planning may need to be personalized.

### Implications for further research

This review systematically analyzed the existing evidence on relation between OLP and PIDs for the first time. Large-scale prospective trials are required to validate the findings.

The results suggest that OLP is not a potential risk factor for PIDs during the 1 to 10-year follow-up period. However, given the relatively small amount of evidence available, the final answer to this question depends on large, well-designed prospective and randomized clinical trials in the future. At present, the proportion of people undergoing implant treatment and the prevalence of OLP worldwide are not high, which may cause difficulty in researching the correlations between these two diseases; therefore, multicenter cooperative clinical research is needed. Additionally, OLP has various clinical classifications, and the prospective study included in this systematic review showed that OLP patients with desquamative gingivitis had a higher frequency of PIM. This suggests that in future studies, subgroup analysis of OLP may be helpful in exploring the associations between OLP and PIDs. Moreover, interventional studies on the effects of OLP treatment on peri-implant status are still lacking; these studies can further elucidate the associations between OLP and PIDs. Molecular biology techniques can also help to explore the microscopic effects of OLP on peri-implant pathophysiological processes at molecular level.

## Conclusions

Available articles regarding the effects of OLP on PIDs remains very limited. Existing evidence does not support OLP as a suspected risk factor for PIDs. Large-scale prospective trials are required to validate the findings.

## Data Availability

The datasets used and/or analyzed during the current study are available from the corresponding author on reasonable request.

## Abbreviations

OLP: Oral lichen planus
PIDs: Peri-implant diseases
RR: Risk ratio
CI: Confidence interval
PIM: Peri-implant mucositis
PI: Peri-implantitis
PRISMA: Preferred Reporting Items for Systematic Review and Meta-analyses
NOS: Newcastle-Ottawa Scale
AHRQ: Agency for Healthcare Research and Quality
